# First report of a Chinese patient carrying Hb Ty Gard: A case report

**DOI:** 10.1097/MD.0000000000031902

**Published:** 2022-11-18

**Authors:** Yuanyuan Pei, Jian Ran, Fengxiang Wei

**Affiliations:** a Central Laboratory, Longgang District Maternity & Child Healthcare Hospital of Shenzhen City, Shenzhen, Guangdong, China.

**Keywords:** capillary electrophoresis, Hb Ty Gard, *HBB*

## Abstract

More than 1300 mutations which lead to abnormal hemoglobin (Hb) have been recorded in the HbVar database. Hb Ty Gard has rarely been reported and has not been reported in China.

**Patient concerns and Diagnoses:**

A 2-year-old Chinese girl was healthy with normal physical development and hematological parameters. Capillary electrophoresis suggested that Hb F increased slightly, while Hb A2 levels were normal. Flow cytometry, fluorescence hybridization, and Sanger sequencing were used to characterize the genotypes. Sanger sequencing detected a heterozygous mutation at codon 124 of the β-globin gene (HBB: c.374 C > A), which was previously reported as Hb Ty Gard in the HbVar database.

**Outcomes:**

We report the first case of HbTy Gard in a Chinese population. In areas with a high incidence of Hb diseases, sensitive detection of Hb components and accurate diagnosis of Hb variation are very important, and the combined application of capillary electrophoresis and gene sequencing can diagnose more Hb variants.

## 1. Introduction

Hemoglobinopathies can be categorized into 2 major categories: thalassemia and abnormal hemoglobin (Hb) levels. Insufficiencies of α- or β-Hb caused by deletion or single nucleotide variants of α- or β-globin genes (HBB) lead to thalassemia. Some mutations in globin genes related to Hb structural isoforms without a dosage effect on Hb lead to abnormal Hb levels. Thalassemia and abnormal Hb levels are widespread in southern China.^[1]^ Severe α- and β-thalassemia are highly focused in prenatal diagnosis because they have a serious impact on the length or quality of life. However, abnormal Hb levels mostly cause a mild phenotype even without clinical symptoms, which is easily missed in prenatal and neonatal screening.

To date, more than 1300 mutations that lead to abnormal Hb levels have been recorded in the HbVar database (https://globin.bx.psu.edu/hbvar/menu.html). Excluding hemolytic Hb (Hb S),^[2]^ abnormal oxygen affinity Hb^[3]^ and thalassemia-like abnormal Hb (such as Hb E),^[4]^ which obvious functional changes have occurred, the phenotype of the carriers of most individuals with other abnormal Hb conditions is normal (https://globin.bx.psu.edu/hbvar/menu.html).

Hb Ty Gard is a rare abnormal Hb caused by an HBB mutation, which has only been reported in France and India, and the reported hematological phenotypes were different.^[5,6]^ Here, we report a case of abnormal Hb Ty Gard in a Chinese patient for the first time, and include the results of complete blood count and Hb analyses.

## 2. Case presentation

The patient was a 2-year-old girl from Shenzhen City, Guangdong Province, China. She was referred to our hospital for routine physical examination in the child’s growth department. The inspection showed normal physical development and hematological parameters (RBC:4.75*1012/L, Hb: 133 g/L, MCV:83fl, MCH:28pg, MCHC: 336 g/L, RDW:12%), and the patient had no previous history of anemia, had not received any blood transfusions, and did not have an abnormal family history. The subject’s guardian agreed to participate in the study and signed an informed consent form. The study was approved by the Ethics Committee of Shenzhen Longgang Maternal and Child Health Hospital (Shenzhen, China).

**Figure 1.**
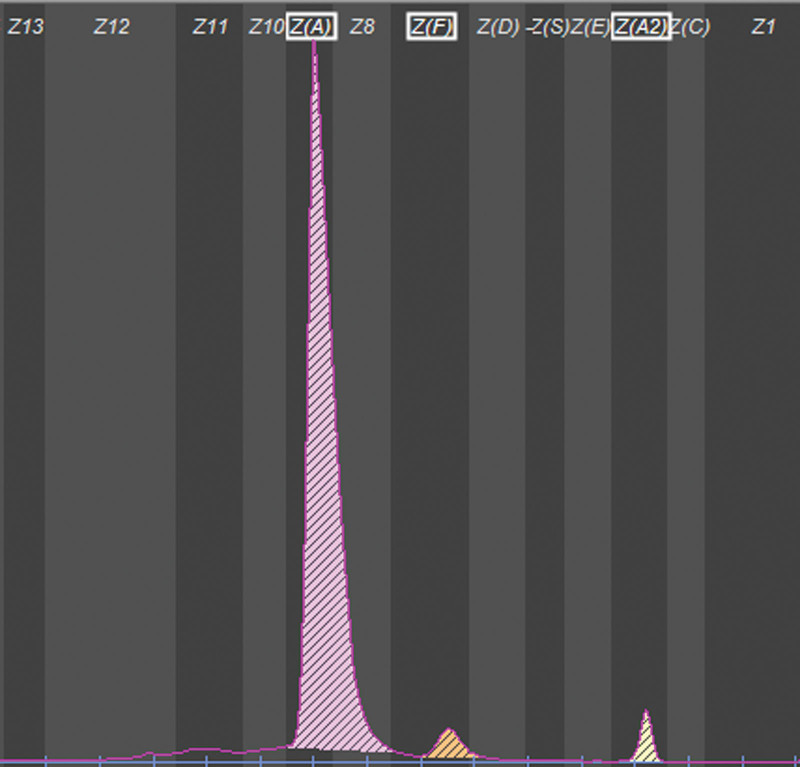
Capillary electrophoresis of Hb Ty Gard with Hb F (3.3%) increased slightly while Hb A2 (2.9%) was normal. Hb = hemoglobin.

**Figure 2.**
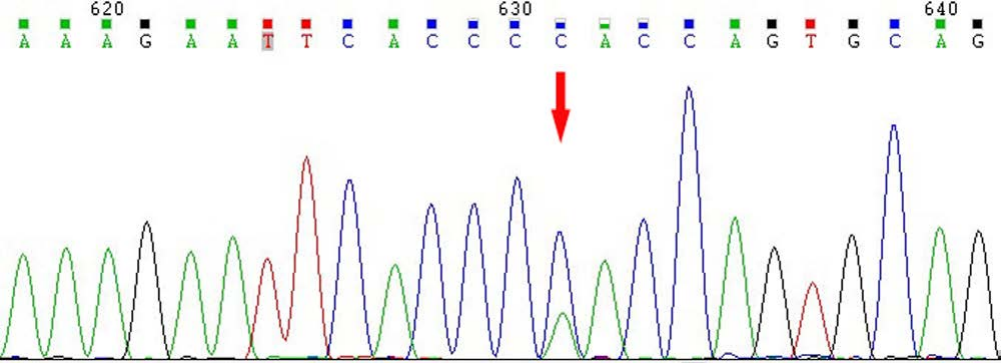
Forward direct DNA sequencing of the *HBB* gene. Arrow indicates the heterozygosity mutation at codon 124 of HBB (*HBB*: c.374c > A) that was previously reported as Hb Ty Gard in the HbVar database. Hb = hemoglobin, HBB = β-globin gene.

Hb analysis was performed using an automated capillary electrophoresis system (Sebia). Capillary electrophoresis revealed 3 peaks, 1 corresponding to adult Hb A (93.8%), 1 corresponding to Hb F (3.3%), and 1 corresponding to Hb A2 (2.9%). The Hb F percentage was slightly higher(normal range 0.5%-2.5%), while that of Hb A2 was normal (Fig. 1). A total of 23 mutations, which were common in individuals from southern China, were routinely measured using PCR combined with flow cytometry, and no abnormalities were found. The HBB was then amplified and sequenced. Sanger sequencing detected a mutation at codon 124 of *HBB* [β 124(H2) Pro > Gln, *HBB*: c.374 C > A] (Fig. 2), which was previously reported as the HbTy Gard in the HbVar database (https://globin.bx.psu.edu/hbvar/menu.html).

## 3. Discussion

To the best of our knowledge, Hb Ty Gard is a rare disease worldwide and has not been previously reported in China. In 1978, Hb Ty Gard was first reported in a 45-year-old French patient with polycythemia. This was not evident based on routine electrophoresis and was only successfully isolated by isofocusing electrophoresis. Functional studies have shown that the oxygen affinity of Hb Ty Gard increases.^[5]^ In 2019, Wahengbam et al^[6]^ found that a 15-year-old Indian boy was a carrier of Hb Ty Gard. The hematological parameters of the boy were normal, and the Hb F level was increased (4.7%). They found that elevated fetal Hb levels compensated for defective β-globin products and significantly reduced the severity of sickle cell anemia.

In the present case, the patient showed only a slight increase in Hb F without any other hematological abnormalities. Whether erythrocytosis occurs with age requires further investigation. Unfortunately, the HBB mutation and hematological parameters of the parents were not obtained.

Several other abnormal Hb isoforms can occur as a result of a mutation at the same position as Hb Ty Gard, including Hb Tende (β124(H2) Pro > Leu, HBB:c.374C > T)^[7]^ and Hb Khartoum (β124(H2) Pro > Arg, HBB:c.374C > G),^[8]^ and the hematological parameters for these are typically normal. Although the substitution of Gln for Pro at β 124(Ha) may weaken the contact between the β chain and the α chain, Hb Ty Gard has a moderately increased affinity for oxygen^[5]^; heterozygotes show no signs of anemia or thalassemia hematological phenotypes. However, the pathogenesis of these variants is poorly understood.

Capillary electrophoresis can separate Hb components with different surface charges and calculate the proportion of each Hb component using the different swimming rates of charged molecules in alkaline buffer. It has the advantages of a high resolution, good repeatability, fast detection speed, and less interference. The combined application of capillary electrophoresis and gene sequencing can be used to diagnose abnormal Hb isoforms.^[9]^

## 4. Conclusion

In the present report, a 2-year-old girl of Chinese ethnicity with normal physical development and hematological parameters and Hb Ty Gard is described for the first time. As these abnormal Hb isoforms may have adverse effects on individuals with age, in areas with a high incidence of Hb attributable diseases, sensitive detection of Hb components and accurate diagnosis of Hb variants is very important.

## Author contributions

**Funding acquisition:** Fengxiang Wei.

**Methodology:** Jian Ran.

**Resources:** Fengxiang Wei.

**Writing – original draft:** Yuanyuan Pei.
